# ANCA-Associated Vasculitis in a Patient Presenting With Rapid Progressive Glomerulonephritis

**DOI:** 10.7759/cureus.20227

**Published:** 2021-12-07

**Authors:** Arminder Singh, Stephanie Everest, Lam Nguyen, Mark Kasari

**Affiliations:** 1 Internal Medicine, Cape Fear Valley Medical Center, Fayetteville, USA; 2 School of Medicine, Campbell University School of Osteopathic Medicine, Lillington, USA; 3 Nephrology, Cape Fear Valley Medical Center, Fayetteville, USA

**Keywords:** anca-associated vasculitis, anca-positive vasculitis, rituximab, kidney failure, pauci-immune crescentic glomerulonephritis

## Abstract

Antineutrophil cytoplasmic antibody (ANCA)-associated vasculitis (AAV) is a rare disease distinguished by the presence of circulating ANCA along with inflammation and destruction of primarily small blood vessels. AAV includes granulomatosis with polyangiitis (GPA), microscopic polyangiitis (MPA), and eosinophilic granulomatosis with polyangiitis (EGPA). Overall, AAV occurs more frequently in Caucasian populations with an approximate incidence of 20 per million per year in Europe and North America. This report presents a case of a 70-year-old female with a history of interstitial lung disease who was hospitalized due to markedly reduced renal function and eGFR within the range of end-stage renal disease on admission. The patient tested positive for perinuclear (p)-ANCA, also known as myeloperoxidase (MPO)-ANCA. The patient was subsequently started on hemodialysis and induction therapy of cyclophosphamide and methylprednisolone for glomerulonephritis secondary to p-ANCA vasculitis. The patient was discharged with improved renal function, and she was expected to follow up with nephrology for maintenance therapy to prevent future relapse. This report demonstrates a case of p-ANCA-positive glomerulonephritis treated with cyclophosphamide and methylprednisolone and discusses the current treatment guidelines for glomerulonephritis secondary to p-ANCA vasculitis.

## Introduction

Antineutrophil cytoplasmic antibody (ANCA)-associated vasculitis (AAV) is a rare autoimmune disorder of small to medium blood vessels that may present as renal-limited disease or cause dysfunction of multiple organ systems. AAV includes a wide spectrum of disorders that are unfortunately associated with high morbidity and mortality. In the literature, ANCA vasculitides are further classified by antibody target protein and staining patterns, specifically antibodies that target myeloperoxidase (MPO-ANCA or perinuclear (p)-ANCA) or proteinase 3 (PR3-ANCA or c-ANCA). The various clinicopathologic manifestations of the disease may also be classified as granulomatosis with polyangiitis (GPA), microscopic polyangiitis (MPA), or eosinophilic granulomatosis with polyangiitis (EGPA) [1]. Renal dysfunction may be seen in up to 70% of patients with GPA and greater than 90% of patients with MPA, making renal involvement the most common clinical finding of ANCA-mediated injury, followed by disease of the upper and lower respiratory tract [2]. The major renal pathology associated with AAV is pauci-immune crescentic glomerulonephritis. The treatment of AAV most often includes a regimen of cyclophosphamide and glucocorticoids, as well as immunomodulatory therapies that have been the focus of recent study. However, treatment continues to remain a challenge due to high rates of infection and disease relapse, and a universal consensus on the optimal treatment regimen has yet to be established [3].

In this report, we discuss the clinical presentation, medical management, and outcomes of a patient hospitalized due to p-ANCA-associated vasculitis and pauci-immune crescentic glomerulonephritis.

## Case presentation

A 70-year-old female with a history of hypertension, diastolic congestive heart failure, atrial fibrillation, interstitial lung disease, recurrent pneumonia, subacute thyroiditis, and obesity was hospitalized for a rapid change in kidney function over the previous two months. The patient’s initial workup was remarkable for normocytic anemia, 3+ hematuria, proteinuria, decreased eGFR of 4.7 mL/minute/1.73 m^2^, and increased blood urea nitrogen (BUN) of 92 mg/dL and creatinine (Cr) of 7.87 mg/dL, demonstrating a significant decline from her baseline eGFR of greater 60 mL/minute/1.73 m^2^, BUN of 9 mg/dL, and Cr of 0.70 mg/dL one year prior. The patient had discontinued Lasix one month prior due to her kidney function and reported that she had not been compliant with taking any of the medications prescribed to her at the time. The patient stated that she had experienced constant nausea and occasional non-bloody vomiting over the past three weeks. She also endorsed recent fatigue, generalized weakness, worsening bilateral lower extremity edema, exertional dyspnea, decreased urine output, and hematuria. Chest X-ray revealed reticulonodular interstitial markings without significant change from prior scans and consistent with her previous diagnosis of interstitial lung disease. A renal ultrasound was negative for obstructive uropathy, and nephrology was consulted for further workup.

**Figure 1:**
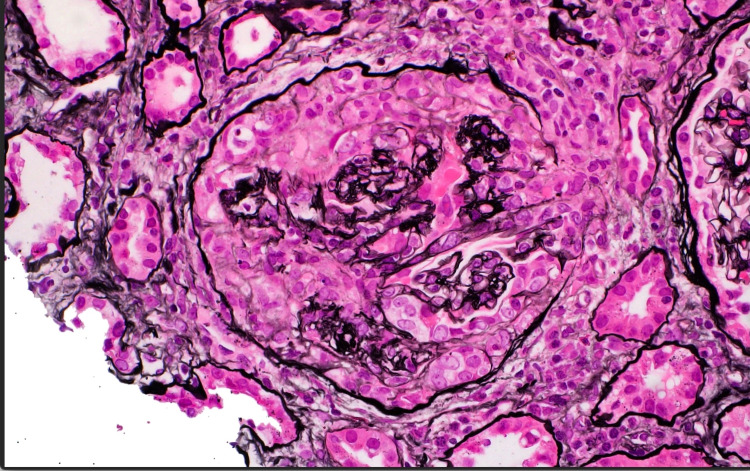
Biopsy with Jones methenamine silver stain demonstrating the crescentic features consistent with ANCA vasculitis

**Figure 2:**
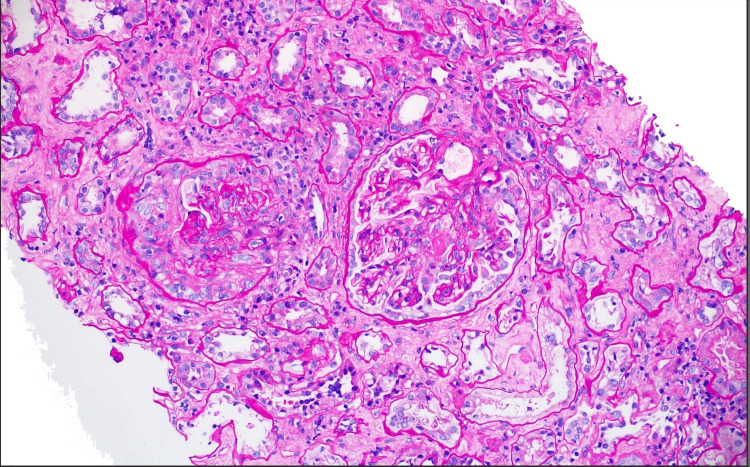
Biopsy with PAS stain demonstrating the crescent formation consistent with ANCA vasculitis

**Figure 3:**
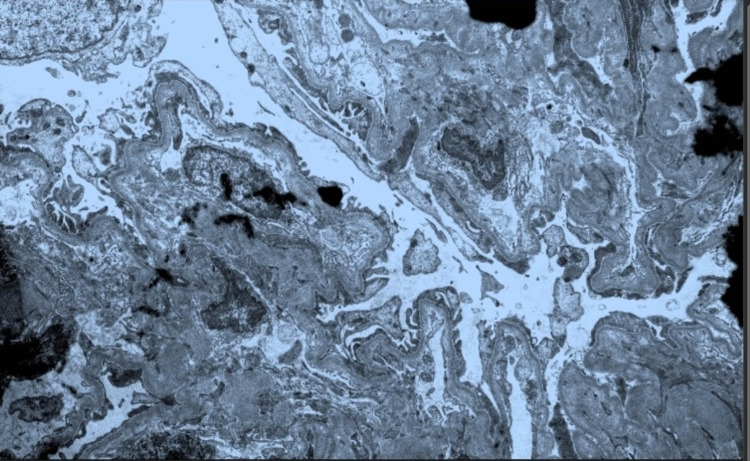
Electron microscopy demonstrating no diagnostic immune complex-type deposits with severe foot process effacement consistent with pauci-immune crescentic glomerulonephritis

Due to the rapid decline in her kidney function, further investigation was initiated to identify the etiology with the differential diagnosis of nephritic syndrome, nephrotic syndrome, and vasculitis. A permacath was placed, and hemodialysis was started immediately. Serology for ANCA was ordered and came back positive for p-ANCA. A renal biopsy was also obtained, and the results revealed pauci-immune necrotizing, crescentic glomerulonephritis, focal acute tubulointerstitial nephritis, interstitial fibrosis, and moderate tubular atrophy (Figure 1, 2, 3). Following the confirmation of glomerulonephritis secondary to p-ANCA per renal biopsy and laboratory results, the patient was started on induction therapy of one dose of cyclophosphamide of 700 mg and three days of methylprednisolone 1 g. The patient was subsequently discharged with improved eGFR of 11.1 mL/minute/1.73 m^2^, and she was scheduled to follow up with nephrology outpatient for further hemodialysis, continuation of cyclophosphamide, and 30 days of oral prednisone.

## Discussion

ANCA-associated vasculitis is a rare heterogeneous disease involving necrotizing inflammation of small to medium blood vessels and possible granulomatous formation. Effects are systemic, and radiographic imaging and urinalysis may aid in determining organ involvement and disease severity. Findings of AAV may include scleritis, sinusitis, pulmonary fibrosis, alveolar hemorrhage, glomerulonephritis, mononeuritis multiplex, cutaneous vasculitis, and arthritis. Previous reports have shown that less severe disease that is limited to the upper airway frequently progresses to vital organs, and severe presentations of the disease may be preceded by a milder prodrome of flu-like illness [4]. Sinus involvement and granuloma formation are considered the hallmarks of granulomatosis with polyangiitis and are usually PR3-ANCA positive, while microscopic polyangiitis is associated with interstitial lung disease and is most often MPO-ANCA positive [5].

Recent studies suggest restructuring the AAV subgroups in order to reflect clinical prognosis and guide treatment. These studies demonstrated that PR3 antibodies and lack of renal involvement are associated with a lower risk of severe organ damage but a twofold higher risk of relapse [4]. Conversely, MPO antibodies and renal involvement are associated with a higher risk of severe organ damage and a lower risk of relapse. MPO antibodies were also associated with a higher incidence of severe infection [4,5]. These findings suggest that stratifying AAV based on PR3 and MPO antibody presence is crucial to developing appropriate treatment regimens. Personalized treatment for patients involves balancing the risk of disease relapse with the risk of medication toxicity and adverse effects. Studies evaluating AAV treatment outcomes have demonstrated that deaths after the first year of treatment are most commonly due to infection, malignancy, and cardiovascular events rather than active vasculitis. For this reason, prolonged immunosuppressant use in the treatment of AAV is controversial, and immunomodulatory therapies have been under investigation as possible alternatives to standard therapy [4].

Guidelines set forth by the European League Against Rheumatism (EULAR) in 2015 recommended the use of high-dose glucocorticoids in combination with cyclophosphamide or rituximab for the induction of remission in patients with organ-threatening AAV. In those with non-organ-threatening diseases, a regimen of glucocorticoids plus methotrexate or mycophenolate mofetil is considered the less toxic alternative [6]. The comparison of immunotherapies has been a major focus of AAV research. In the Rituximab in ANCA-associated Vasculitis (RAVE) Trial, remission rates were compared at six months after the completion of a glucocorticoid taper in 197 patients with AAV who received rituximab or cyclophosphamide plus azathioprine. No significant difference was found between the two groups overall; however, rituximab was superior in remission rates among those with relapsing disease at baseline and those with PR3-ANCA-positive vasculitis. Rituximab was also associated with a decreased instance of pneumonia when compared with the cyclophosphamide-azathioprine group [7]. Clinically, the use of rituximab may be limited by its cost. There is currently limited evidence to support the combined use of rituximab and cyclophosphamide in order to forgo the prolonged use of high-dose glucocorticoids. Conflicting evidence exists on the duration of glucocorticoid administration, and a few studies propose that timing should vary based on the patient’s risk of relapse [4].

Establishing the standard therapy discussed has allowed initial remission rates of as high as 90% in patients with AAV, but preventing disease relapse remains a major challenge as less than a third of patients remain in relapse after 10 years [7,8]. For maintenance of remission, the EULAR recommends the use of low-dose glucocorticoids with either azathioprine, rituximab, methotrexate, or mycophenolate mofetil [6]. Studies comparing the long-term efficacy of these medications are complicated by the wide-ranging manifestations and rarity of AAV. Available evidence suggests that mycophenolate mofetil is appropriate in less severe diseases and MPO-ANCA-positive AAV. A 2020 review of the literature reported one randomized control trial (RCT) in which azathioprine and methotrexate showed similar rates of relapse-free survival and adverse events, while two RCTs demonstrated that rituximab was superior to azathioprine in preventing disease relapse [4]. Additional research is needed to further compare the efficacy of these medications in the remission induction and maintenance of PR3- versus MPO-positive ANCA vasculitis. Further study is also necessary to establish an appropriate dose and duration of glucocorticoid therapy for proper management while weighing the adverse effects of long-term use.

The patient presented in this case report had AAV that was positive for MPO antibodies complicated by rapidly progressive glomerulonephritis and end-stage renal disease. Of note, this patient had a prodromal flu-like illness of malaise, dyspnea, nausea, and vomiting that began weeks prior to seeking emergent medical care. The year prior to her onset of renal disease, she was hospitalized multiple times for recurrent pneumonia and diagnosed with interstitial lung disease, suggesting a significant pulmonary involvement related to her AAV. However, a conformational lung biopsy was not performed. Given this patient’s clinical presentation and laboratory findings, her AAV is most consistent with microscopic polyangiitis. As such, this patient’s case adds to the current literature establishing an association between interstitial lung disease and the onset of MPO-ANCA-positive vasculitis. Unfortunately, evidence has shown that the presence of MPO antibodies and interstitial lung disease are each associated with poor prognosis, as well as higher rates of infection on immunosuppressant therapy when compared with controls [9].

## Conclusions

ANCA-associated vasculitis is a rare systemic disease that presents with a wide spectrum of clinicopathologic manifestations, making it a challenging disease to establish universally accepted guidelines for remission induction and maintenance. To our knowledge, there is currently no difference in the recommended standard treatment for those with ANCA vasculitis and interstitial lung disease, and further research is needed in this specific population. This case study documents the findings and management of a hospitalized patient diagnosed with p-ANCA-positive vasculitis presenting with pulmonary and renal involvement.
